# Comparative Studies of the Effectiveness of Rotational and Vibratory Machining

**DOI:** 10.3390/ma19081554

**Published:** 2026-04-13

**Authors:** Damian Bańkowski, Piotr Młynarczyk, Wojciech Depczyński

**Affiliations:** Department of Materials Science and Materials Technology, Faculty of Mechatronics and Mechanical Engineering, Kielce University of Technology, al. 1000-lecia P.P. 7, 25-314 Kielce, Poland; piotrm@tu.kielce.pl (P.M.); wdep@tu.kielce.pl (W.D.)

**Keywords:** fine machining, rotational machining, vibratory machining, MRR, geometric structure of the surface, planned experiment

## Abstract

Container machining plays a key role in the finishing of workpieces. The aim of this article was to compare the effectiveness of vibratory and high-speed rotational machining. Mass loss and selected changes in surface geometric structure parameters were assessed. To obtain a porous structure, the samples were prepared by sandblasting. The novelty of this work is the use of high rotational speeds for rotational machining and the use of a planned experiment to limit the number of samples. The innovative nature of the comparison of vibratory and high-speed rotational machining allowed the development of mathematical models of the influence of process parameters on the final results. A two-factor planned experiment with five levels of process variables was used to investigate a wide range of process input variables. Based on the RSM response surface, mathematical models of changes in mass losses MRR, arithmetic mean surface roughness Ra, maximum height of the highest elevation (peak) of the roughness profile Rp, and surface skewness Ssk as a function of input parameters were developed. Working containers with a volume of 25 dm^3^ were used for the tests, and the test material was samples made of PA38/EN AW 6060 aluminum. Studies have shown that, for similar machining times, greater MRR changes were achieved with rotary machining. Rotary machining using the same machining media and similar machining times was characterized by up to 15% greater MRR than vibratory machining after 75 min of container machining. The reason for this high efficiency is the use of high rotational speeds. Comparing the effectiveness of reducing surface geometric structure parameters between rotational and vibration machining processes depends primarily on the machining time. The work proves that the use of rotational machining and high rotational speeds allows for shorter machining times compared to vibration machining.

## 1. Introduction

Loose-body machining involves the continuous and free action of media in the form of shaped parts on the workpieces. It is also referred to as container machining. These processes are based on the mechanical impact of the working charge on the workpieces. The origins of loose abrasive processing date back to the first attempts at barrel processing. The working charge consisted of natural materials such as stones and sand. The goal of the processing was to smooth surfaces and round edges. Only with time and technological progress did natural abrasives begin to be replaced with dedicated abrasive and polishing media. The efficiency of the first rotary machine tools was insufficient, so a vibratory motion was implemented to drive the working containers. The combination of rotational motion and vibrations contributed to the intensification of interactions between the workpieces and the working load media.

Rotational machining is a container machining process in which the work container rotates. Containers can be open or closed, oriented horizontally, vertically, or mounted at an angle. The workpieces can also be placed freely and move with the workpiece, or they can be fixed, and the movement of the workpiece will affect the other workpieces [1].

The first patent for rotary-drum finishing using an oscillating mechanism was filed in Germany in 1936 [2]. And the first patents for vibratory finishing machines of the ladle and bowl types were filed in 1960 in the USA and Great Britain, respectively [3,4]. Since then, vibratory finishing processes have been widely used in industry. However, there is little scientific literature describing the fundamentals of vibratory finishing, such as machine design and the metal removal mechanism.

Vibratory machining is a type of abrasive machining process by mixing workpieces with appropriately selected media: abrasive or polishing material in a vibrating container (tumbler) [5,6]. A wide range of materials dedicated to vibration machining is available. The media differ primarily in type, i.e., we can distinguish polyester, ceramic, porcelain, metal, or natural media such as corn cobs, nut shells, or even wood. Media can come in various sizes, from a few millimeters to as much as 50–60 mm or more. Furthermore, media come in various shapes, including those with sharp edges, such as pyramids, cylinders, truncated cylinders, stars, satellites, and those with rounded edges, designed for polishing and brightening processes, such as spheres. The shape and material of the media influence the processing results. Therefore, it is important to select the correct media for the workpiece. Furthermore, vibratory machining can be performed dry or wet, with the addition of fluids or dedicated pastes. The low operator effort required for the process contributes to its widespread use. Vibratory machining is increasingly used for surface deburring, edge rounding, polishing, and surface brightening. The design of the vibrating device directly influences the machining results. Among vibratory processing devices, we can distinguish circular, oval, trough, spiral, spatial, or plane vibration devices.

The most important process variables are the size and type of the machine, the type, size, and shape of the abrasive media, the amount of the working charge and its ratio to the number of workpieces, the amount of supporting fluids and its ratio to the amount of other media, and the processing time [7].

The characteristics of vibration machining processes and the formation of layers as a result of the processes have been the subject of many studies [5,6].

In the Hashimoto work [1], a mathematical model of a bowl-type vibratory finishing machine was presented, and the dynamic behavior of the mechanical vibratory system under free and forced vibration conditions was analyzed. Hashimoto et al. discussed the influence of the main parameters of the proposed model on the efficiency of the vibration machining process.

Karthik et al. [8] used vibration machining to reduce the surface roughness of selectively laser-sintered Inconel 718 alloy components. Experimental results confirm that the surface roughness of laser-sintered and unsintered samples was reduced at a frequency of 75 Hz compared to 50 and 25 Hz. Higher frequencies result in a greater number of reciprocating movements performed by the vibrating device per unit of time. This confirms the correct assumption that increasing the number of impacts will contribute to achieving results in a shorter time.

Wen et al. [9] developed a similarity model in horizontal vibration finishing and derived a calculation formula for the distortion coefficient to correct the prediction results. In this paper, the influence of vibration parameters on the characteristics of particle velocity and normal force under different vibration parameters was investigated using DEM simulation. The Discrete Element Method (DEM) is a numerical framework for simulating the behavior of interacting discrete bodies, allowing the observation of finite displacements and the recognition of various contacts or interactions between them. It facilitates the definition of particle interactions and motion, allowing easy coupling to other particle-based methods.

Also, in [10], Hao et al. compared the final effects and mechanisms of normal forces in the vibratory finishing process. It was found that the measured normal force has the same frequency-multiplication force characteristics as the simulated force, and the simulation results can reflect the finishing pattern. The normal action of particles on the surface, or normal force, can cause the cavity surface to form a lower roughness faster than tangential action. It is possible to control the contact angle of the abrasive media with the workpieces, which directly affects the machining effects.

In the work by Wang [11], a novel three-axis wireless force sensor was proposed for measuring the forces of vibratory surface finishing. The sensor does not have any wiring that would affect the trajectory of the workpiece in the machine container. Experimental results [11] show that normal impact forces between the medium and the workpiece dominate in vibratory surface finishing. The average normal forces on the front surface of the workpiece are larger than those on the side and rear surfaces of the workpiece. The mass and shape of the medium have a significant influence on the vibratory surface finishing forces, which in turn have a significant influence on the roughness and hardness of the workpiece surface.

The use of vibratory machining for polishing tungsten carbide coatings deposited by chemical vapor deposition was investigated by Micallef et al. [12]. The study demonstrates that vibratory machining can be used to finish surfaces with hardnesses above 1300 HV without causing any loss of coating thickness. Vibratory machining for 4 h resulted in a reduction in the Ra parameter by 0.2 μm. Scanning observations show that the dominant polishing mechanism was abrasive wear, which allowed for an overall improvement in surface finish but with the formation of micro-wear marks.

In the work of Bańkowski [13], it was pointed out that vibration treatment enables an increase in hardness. Using steel media, an increase in hardness was obtained for brass from about 70 HV_0.02_ to about 120 HV_0.02_ after nearly 1.5 h of vibratory machining. Moreover, the paper found that the vibratory machining time has a much greater effect on reducing the arithmetic mean surface roughness, Sa, than the vibratory machining frequency.

The main scientific contribution of this work is a comparison of the effectiveness of vibratory machining with high-speed rotational machining. Previous research has focused on rotational machining at relatively low speeds, below the critical speed. Furthermore, the literature lacks studies on the selection of process parameters depending on the desired changes in mass loss or changes in surface geometric structure parameters.

**Figure 1:**
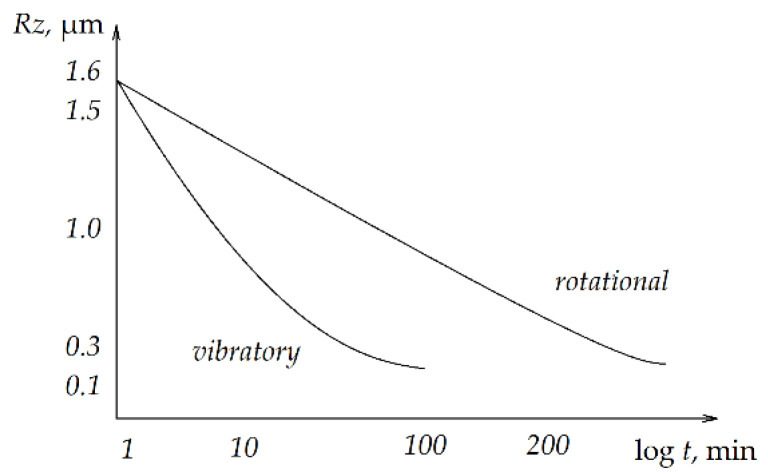
The change in the surface roughness of C10 steel samples depending on the finishing time in container smoothing machines was developed on the basis of [14].

The aim of the research in this article was to compare the effectiveness of container machining processes. The novelty lies in determining the mathematical relationships between the influence of basic factors in the rotational and vibratory machining processes on the effects. Based on the conducted research, potential users can utilize the developed mathematical models to determine the required machining times, rotational speeds, or vibration frequencies to achieve desired values for surface geometric structure parameters or mass losses. The literature provides examples of authors who have addressed the issue of comparing changes in height parameters. The previous research results of Filipowski [14] suggested that vibration machining allows for a reduction in surface roughness Rz much faster than in the case of rotational machining, as shown in Figure 1. However, the mentioned study does not provide any guidance on the selection of process parameters in order to reduce the values of geometric structure of the surface parameters to the assumed value or mass losses. From Figure 1, it can be seen that the reduction in the Rz parameter occurs much faster in the case of vibratory machining than in the case of rotational machining.

## 2. Materials and Methods

**Figure 2:**
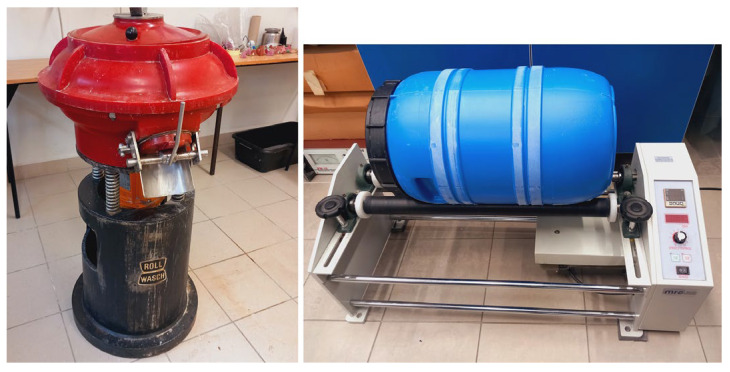
The Rollwasch Rollwasch SMD-25-R device for vibratory machining and MRC Scientific mill instrument for rotational machining.

To compare the two container machining methods, an MRC RJM102 (EnviSense, Lublin, Poland) Scientific ball mill instrument for rotational machining was used. Controlling the rotational speed of the drive motor allowed for setting the desired rotational speed of the working container. The vibrating device used was a Rollwasch Rollwasch SMD-25-R (Rollwasch Italiana S.p.a., Albiate (MB), Italy). The experimental setup is shown in Figure 2. The capacity of both tanks was 25 dm^3^.

The selected test material was a PA38/ EN AW 6060 aluminum alloy (Grupa Kęty S.A., Kęty, Poland) in the form of tubes with a diameter of 28 mm, a length of 50 mm, and a wall thickness of 1 mm. To assess the mass loss of the processed samples and changes in the geometric structure of the surface, the samples were subjected to a sandblasting process using coarse electrocorundum as the medium. The sandblasted samples were characterized by a developed surface, different from the delivered condition in the form of a pipe after plastic processing.

The media used for the tests were PB14KT polyester media (Rollwasch Italia-na S.p.a., Albiate (MB), Italy) in the form of cones with a diameter of 14 mm and a height of 10 mm. Based on previous experience, container processing was carried out with the working tank filled with 10 dm^3^ and approximately 150 mL of MEL 100-A22 container processing support fluid (Rollwasch Italiana S.p.a., Albiate (MB), Italy). The purpose of using cutting fluids is to flush out abrasive products and protect the workpieces from corrosion. Furthermore, the addition of fluids reduces dust formation and changes the consistency of the working charge.

In order to compare both processes, it was decided to focus on the functional characteristics of the processed parts, i.e., mass loss and changes in the geometric structure of the surface. The test samples were appropriately labeled and each time cleaned in an ultrasonic bath and weighed before and after processing. Mass loss tests were performed using a Sartorius CPA124S-0CE (Sartorius AG, Goettingen, Germany) scale with a precision of 0.0001 g. Prior to the commencement of the experimental procedure, the scale was subjected to a process of calibration. Material Removal Rate (MRR) [15] was determined for the tested samples. MRR is the amount of material removed per unit of time, e.g., in cubic millimeters per hour, during a machining process such as cutting or grinding. A high MRR indicates high process efficiency, while a low MRR may indicate inefficiency or the need to adjust machining parameters to prevent excessive tool stress and quality degradation.

The material removal rate was determined based on the formula below:

$$MRR=\frac{\left(m_{2}-m_{1}\right)}{t\cdot \delta_{Al}}\;\left[\frac{{mm}^{3}}{h}\right]$$

where m_1_ and m_2_ were the mass of the sample before testing and after machining, respectively, *t* was the machining time in hours, and the δ density of aluminum was assumed to be 2.7 g/cm^3^.

**Figure 3:**
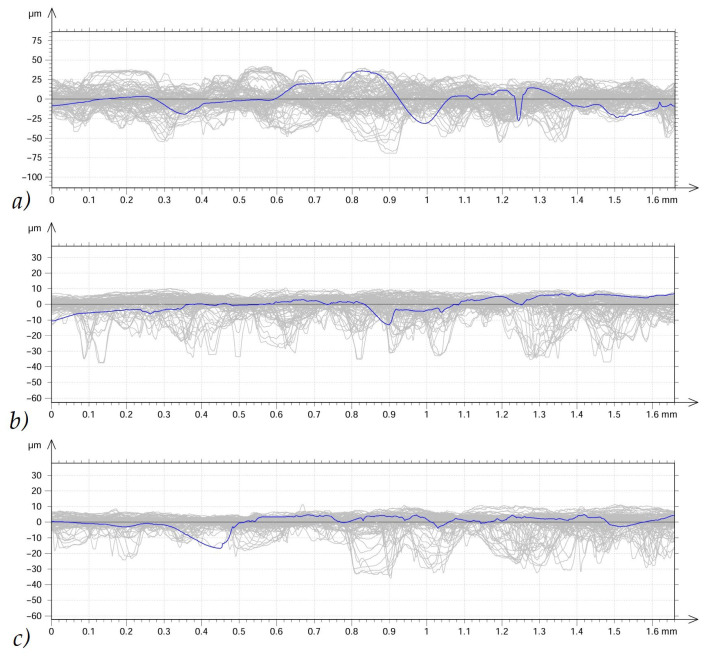
Outline view of 100 roughness profiles (gray) and average roughness profile outline (blue) of (**a**) sample after sandblasting, (**b**) sample subjected to 75 min of vibration treatment, (**c**) sample subjected to 75 min of rotational treatment.

Each sample was then subjected to surface analysis using a Taylor–Hobson Talysurf CCI Lite optical profilometer (Taylor–Hobson, Leicester, UK). Surfaces of 1.6 × 1.6 mm from the middle part of the samples were analyzed. First, the surface curvature was removed, allowing the examined cylindrical surfaces to be visualized as a plane. Next, the unmeasured points were supplemented. A 0.8 mm Gaussian filter was used to calculate the amplitude and height indices of the geometric surface structure. To determine the mean values of linear parameters (R), 100 profiles were collected from the tested 1.6 × 1.6 mm surfaces, and the values were averaged. Surface roughness measurements are often characterized by significant variability at individual sample locations. Therefore, the data summarized in this article refer to average values from 100 measurements on individual samples. Using a sample after sandblasting only, i.e., without container processing, Figure 3 shows an example of a collected series of 100 linear measurements and the determined average line of the surface roughness profile. TalyMap Platinum (software v 5.1.1.5374, Taylor Hobson, AMETEK, Inc., Berwyn, PA, USA) software automatically allows for the determination of mean values and measurement errors, such as standard deviation. The following parameters of the geometric structure of the surface were selected for the analysis and comparison of rotational and vibratory machining:

- Ra is the arithmetic mean deviation of the roughness profile from the mean line. Ra is the basic parameter determining the surface quality, defined as the arithmetic mean of the absolute values of the roughness profile deviations from the mean line on the selected measurement section [16].
- Rp is the maximum height of the highest peak of the roughness profile, measured from the mean line within the elementary section. This is a key metric for analyzing surface wear and coatings. Along with Ra (arithmetic mean) and Rz (roughness height), it helps control the geometry of machined parts [17].
- The surface skewness Ssk is a 3D (area) surface roughness parameter that determines the skewness (asymmetry) of the surface roughness distribution relative to the mean plane [18]. The Ssk parameter indicates whether the surface consists primarily of peaks (peaks) or valleys (valleys). Ssk values close to zero indicate that the height distribution is symmetric about the mean plane (e.g., a sinusoidal profile). Ssk values greater than zero (positive skewness) indicate a surface containing mostly sharp peaks and bumps protruding above the mean plane. On the other hand, Ssk values less than zero (negative skewness) indicate a surface consisting mainly of valleys/depressions, and the peaks are flattened (typical of lapped or finished surfaces).

**Figure 4:**
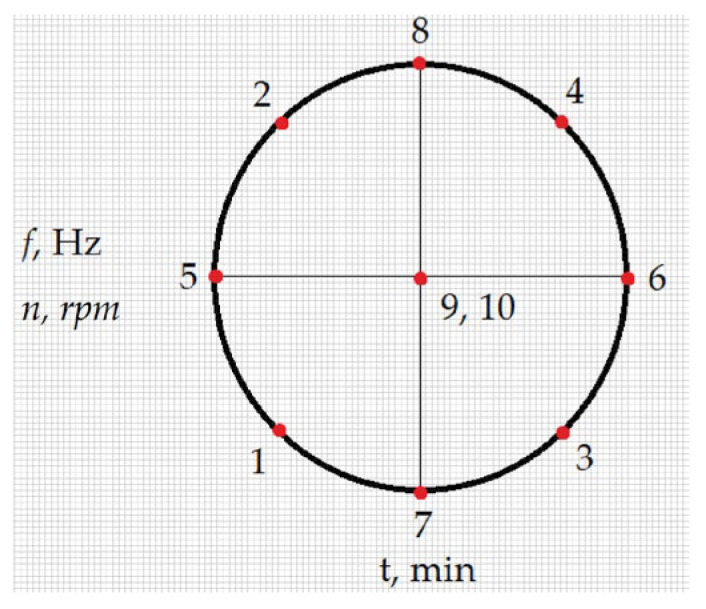
Measurement points of the planned experiment are placed in an orthogonal system.

A two-factor, five-level planned experimental design was used for the study. The diagram of the variable factors, the levels of the study and points from 1 to 10 indicate measurement cases are presented in Figure 4. The choice was supported by the fact that container machining processes are most often based on controlling the machining time and, respectively, in the case of container machining, the rotational speed of the container and, in the case of vibration machining, the frequency. In turn, factor analysis at 5 levels allows for obtaining more information from research than in the case of research at 3 levels of variation [16]. Statistica version 10 64-bit software (TIBCO Software Inc., Tulsa, OK, USA) was used for calculations.

The following input factors were selected for the vibration and rotational machining process:

- Machining time *t*—values of 5, 15, 40, 65, and 75 min, corresponding to the code values −1.414; −1; 0; 1; and 1.414
- Vibration frequency *f*—30, 33, 40, 47, and 50 Hz, corresponding to the code values −1.414; −1; 0; 1; and 1.414
- Rotational speed *n* of the rotational tank—11.2, 20.8, 54.4, 73.6, and 81.6 rpm, corresponding to the code values −1.414; −1; 0; 1; and 1.414

## 3. Results and Discussion

One of the first to attempt to model vibration machining was Hashimoto. In [19], the basic principles of vibration machining were described, and a mathematical modeling approach was proposed that allows for the prediction of surface roughness and stock removal. The adequacy of the modeling approach was compared with experimental results.

The authors of [20] investigated the influence of the contact force and speed between the abrasive material and the workpieces on the material removal rate and surface roughness. The work shows that the size of the abrasive material and the rotational speed of the driving motor had the greatest influence on material removal. The work confirms that rotational speed will be the factor with the greatest influence on the effects of container processing, particularly on mass loss. The novelty of the conducted research is the demonstrated effect of processing time and device rotational speed on changes in surface geometric structure parameters, which was not observed previously in Lachenmaier’s work [20].

The reduction in SGP parameters, including height parameters, accompanying surface treatment is desirable and is one of the main goals of implementing container treatment processes. In [21], the authors observed that the reduction in peak height was accompanied by a reduction in valley depth, which indicates plastic movement of material from peaks to valleys. Furthermore, the study [21] confirmed that saturation of the roughness parameter Ra was observed with process time. Saturation can be interpreted as the time after which the abrasive media is no longer able to abrade the peaks or plastically deform to cause the valleys to fill.

**Figure 5:**
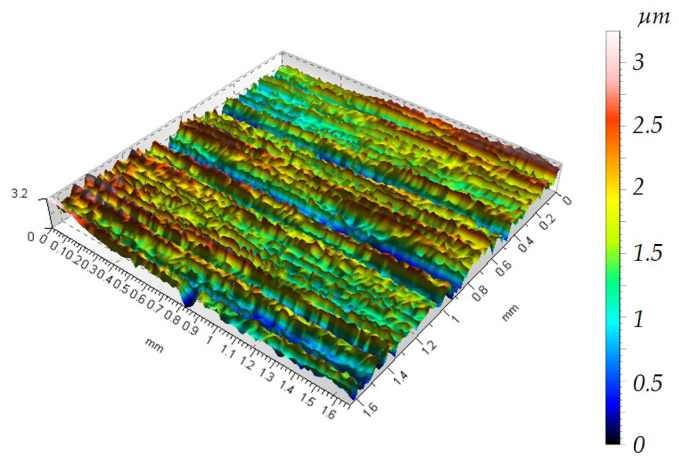
Surface topography pipes in their original condition, Ra = 0.51 µm.

Uhlmann et al. [22] prove that the material removal rate during the transition period of vibratory finishing is strongly dependent on the initial surface roughness. Furthermore, the authors of [22] emphasize the advantage of abrasive media with edges over round media in removing sharp irregularities. An aluminum tube with an isotropic structure was used in this study, as shown in Figure 5.

**Figure 6:**
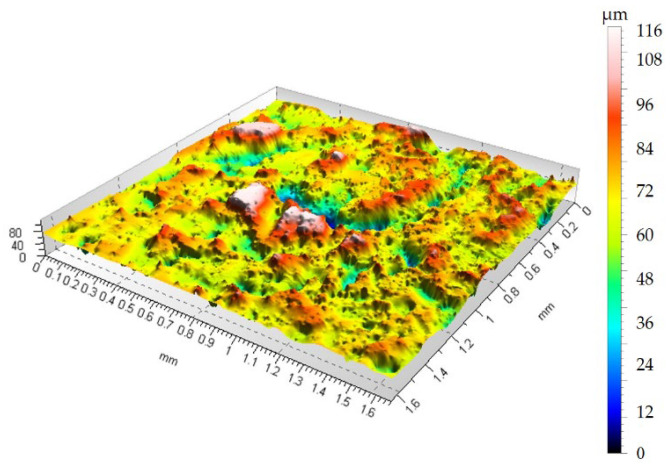
Surface topography after sandblasting, Ra = 8.54 µm.

Before the rotational and vibratory machining tests, the sample surfaces were subjected to a sandblasting process to develop the surface and increase its roughness. This surface preparation will allow for the observation of changes in the height of peaks, valleys, and other parameters of the surface’s geometric structure. The topography of the sample after sandblasting is shown in Figure 6.

**Figure 7:**
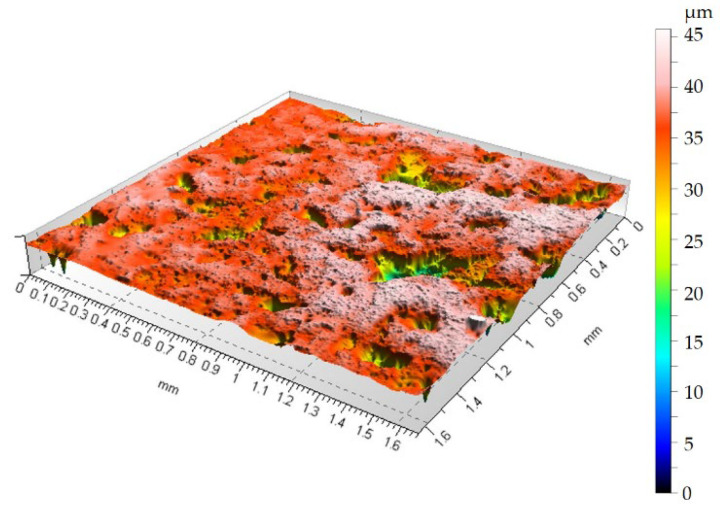
Surface topography after rotational machining, Ra = 2.33 µm.

**Figure 8:**
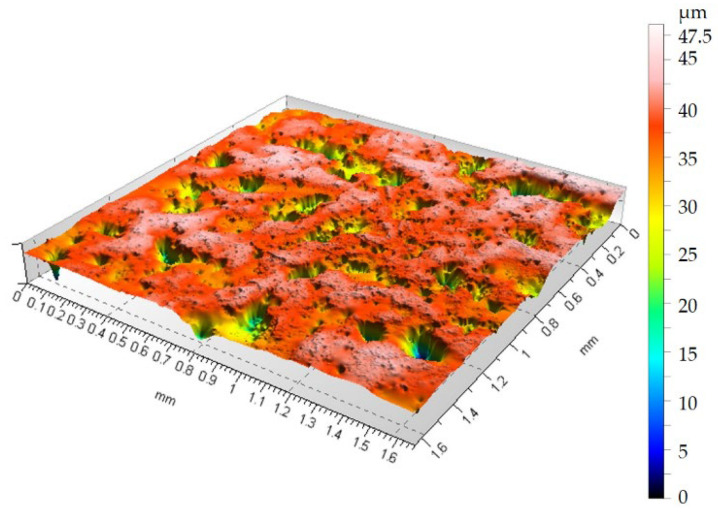
Surface topography after vibratory machining, Ra = 2.84 µm.

To demonstrate how container treatments affect surface topography changes after sandblasting, Figure 7 shows the surface topography after rotational treatment, and Figure 8 shows the surface topography after vibratory machining.

Figure 6 shows that the surface has numerous depressions and accompanying hills compared to the sample before sandblasting in Figure 5. Due to the high surface deformation after sandblasting, characterized by a height distribution of over 110 µm between the highest surface peak and the deepest valley, it can be concluded that the 75 min container treatment removes material within the sandblasted layer. As can be seen from Figure 7 and Figure 8, the height maps are still approximately 45 µm high. Therefore, it can be concluded that the 75 min treatment time removes material and smooths the surface within the layer formed during the sandblasting process.

Analysis of Figure 5, Figure 6, Figure 7 and Figure 8 shows that both vibration and rotational machining allow for a reduction in the arithmetic mean deviation of the roughness profile from the mean line Ra from 8.52 µm to approximately 2.5 µm after 75 min of machining. Surface topographies confirm the effect of mechanical abrasion of the peaks of surface irregularities of workpieces by loose shapes. Loose abrasive sandblasting of workpieces with numerous hills on the surface removes the peaks, thereby reducing the values of the amplitude parameters of the surface geometric structure. Analyzing the isometric views of the surface topography at Figure 7 and Figure 8, it can be seen that as a result of container machining, the surface is smoothed of the largest peaks. Furthermore, the obtained SGP parameter in Table 1 values indicate lower values for the case of rotational machining.

Based on the experimental data obtained, mathematical models of changes in MRR, Ra, Rp, and Ssk were developed using Statistica software. Mathematical relationships between process input parameters and the values of the evaluated parameters were possible thanks to the Response Surface Methodology (RSM) method [17].

Attempts were made to fit the response functions of the RSM models using linear functions, a second-degree polynomial, and interactions of input factors [23]. The best fit was obtained for a second-degree polynomial using multiple regression with backward elimination, for a significance level of α = 0.05. Homogeneity of variance was checked for a significance level of α = 0.05. Conclusions were drawn regarding the significance or non-significance of the effect of a specific term of the regression equation. Terms that were not significant from the point of view of statistical inference were rejected, and only significant terms (*p* less than 0.05) were considered [24,25,26,27]. The obtained results were assessed using analysis of variance (ANOVA) with a significance level of 0.05. For each equation, the coefficient of determination R2 and the alternative coefficient of determination R2-Adj were determined. The adjusted version of the R2 coefficient takes into account the number of variables in the model and thus allows for a more conservative assessment of fit.

The sum of squares (SS) in statistics is a measure of variability that describes how much individual observations in a data set differ from each other and from their arithmetic mean. It is the cumulative sum of the squared differences between each data point and the mean. It is used to measure the degree of dispersion of the data. The larger the sum of squares, the greater the variation (dispersion) of the observations around the mean. Applications (ANOVA and regression): It is the basis for calculations in the analysis of variance (ANOVA) and regression analysis, allowing the partitioning of the total variability into parts explained by the model and unexplained (error).

The number of degrees of freedom is the number of independent outcomes minus the number of relationships/parameters linking them. It is most often calculated as the sample size minus the number of estimated parameters (usually N − 1). In this study, the number of replicates, N, was 10, so the number of degrees of freedom was 9.

In statistics, the F-value is the ratio of two variances, primarily used in analysis of variance (ANOVA) and regression to test whether differences between groups are statistically significant. If the null hypothesis is false, the F-ratio should exceed 1. The greater the differences between the compared groups, the larger the F-ratio and the lower the probability that the difference arose by chance.

The *p*-value is the probability value for the null hypothesis, which is compared to the assumed significance level α. For example, if alpha is 0.05, the result is statistically significant when *p* < 0.05. The ANOVA results are presented in Table 2, Table 3, Table 4, Table 5, Table 6, Table 7, Table 8 and Table 9 [28,29].

The analysis of the influence of vibration and rotational machining factors on the variance of the MRR, Ra, Rp, and Ssk models indicates that:

- The amount of material removed per unit of time, MRR, after rotational machining is influenced by the machining time and rotational speed, while in the case of vibratory machining, only the machining time is influenced.
- The arithmetic mean deviation of the roughness profile from the mean line Ra after rotational machining is influenced by time (linearly) and rotational speed (linearly and squared), while after vibratory machining, only time (linearly) influences.
- The changes in the parameter Rp of the maximum height of the highest elevation (peak) of the roughness profile in the case of rotary machining are influenced by time and rotational speed, whereas for vibratory machining, the changes in the parameter Rp depend only on the machining time.
- The changes in the Ssk parameter after rotational and vibratory machining are influenced by the machining time (linearly) and, respectively, the rotational speed and vibration frequency of the container.

The remaining components and their interactions (for the assumed significance level of α = 0.05) are insignificant.

The determined equations for the amount of material removed per unit of time (MRR) and the arithmetic mean of the roughness profile deviation from the mean line (Ra), the maximum surface peak height (Rp), and the surface skewness (Ssk), as well as the correlation and determination coefficients and the corrected coefficient of determination, are given in Table 10.

The obtained equations are characterized by high (above 0.7) and very high (above 0.9) correlation degrees R. Based on the developed equations, potential operators of container-based machines can determine the required values of the rotary and vibration machining process parameters to achieve the desired mass loss, arithmetic mean surface roughness Ra, maximum surface peak height Rp, and surface skewness Ssk. To date, there have been no studies comparing the effectiveness of rotary and vibration machining. The literature also lacks specific recommendations for vibration and rotational machining process parameters.

The calculated percentage influence of individual input factors of the rotary machining process on the variance of the MRR and Ssk models indicates that machining time has an impact. However, changes in Ra and Rp for rotary machining are influenced by machining time and rotational speed. However, considering the percentage influences of individual input factors of the vibratory machining process, only machining time influences the variance of the MRR, Ra, and Ssk models. In the case of the Ssk model for vibratory machining, time and frequency influence the other components and their interactions (for the assumed significance level of α = 0.05) are insignificant.

The effectiveness of rotary machining is determined by the rotational speed of the working container. The literature [30] recommends rotational speeds ranging from 4 rpm for large drums to 40 rpm for small drums. Higher rotational speeds increase the efficiency of rotary machining. The maximum rotational speed limit for rotational machining is expressed by the relationship Equation (1) [31]

(1) $$n_{kr}=42.3\sqrt{D}$$

where D is the diameter of the working container.

A container with a diameter of 0.32 m was used in the experiment, which indicates that the maximum rotational speed should be no more than 24 rpm. The use of higher rotational speeds leads to a situation in which the centrifugal force causes the working charge to adhere to the walls of the tank, thus preventing it from falling freely [30].

The speeds used in the study were 11.2, 20.8, 54.4, 72, and even 81.6 rpm. Using significantly higher speeds than those recommended by Woźniak and Hinz resulted in very high process efficiency. Furthermore, with the correct selection of abrasive media and the amount of cutting fluids [32], no adhesion of the workpiece to the tank walls was observed.

For a fixed consistency of the working charge, i.e., the ratio of abrasive media to cutting fluids, exceeding the maximum speed resulted in the charge no longer flowing under gravity inside the tank. This resulted in the working charge sticking and preventing proper flow kinematics, thus reducing efficiency. The addition of auxiliary fluids reduced friction forces within the working charge, allowing for proper kinematics and flow during the rotation of the working tank.

The dynamics were high enough to compete with vibratory machining. It can be speculated that using even higher rotational speeds will translate into reduced machining efficiency due to changes in the kinematics of the workpiece movement. When designing rotary machining processes, the rotational speed should be carefully selected to avoid the workpiece sticking to the tank walls. Too high rotational speeds will cause the centrifugal force of the tank’s motion to exceed the gravity field’s effects. As observed in the experiment, each case may be characterized by an individual value of the critical rotational speed. It will depend on the size, density, and mass of individual media, the amount of media placed in the working container, and the amount of supporting cutting fluids.

**Figure 9:**
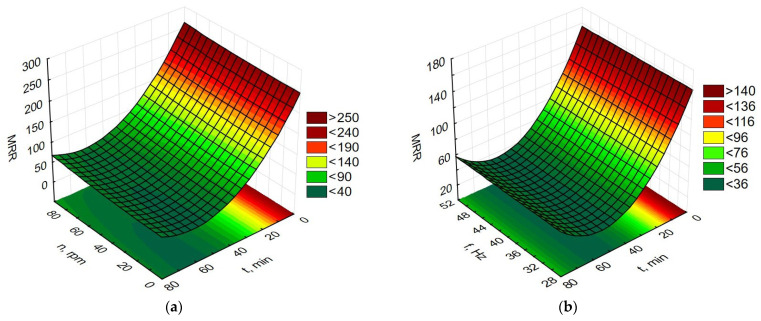
Dependence of MRR changes (**a**) for rotational machining as a function of rotational speed and machining time; (**b**) for vibration machining as a function of frequency and machining time.

**Figure 10:**
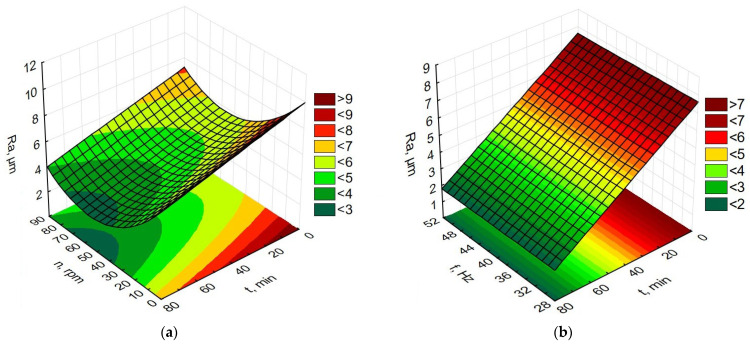
Dependence of Ra changes (**a**) for rotational machining as a function of rotational speed and machining time; (**b**) for vibration machining as a function of frequency and machining time.

**Figure 11:**
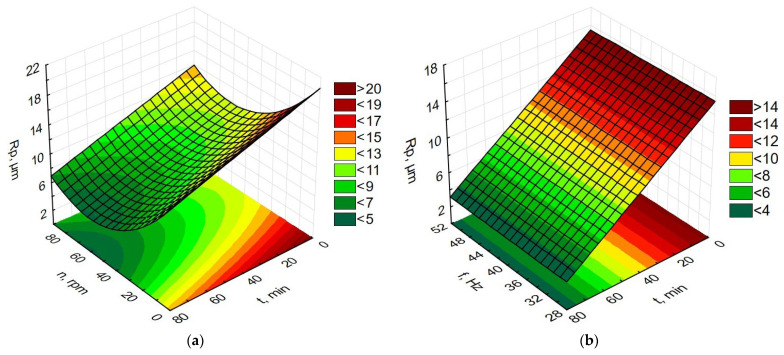
Dependence of Rp changes (**a**) for rotational machining as a function of rotational speed and machining time; (**b**) for vibration machining as a function of frequency and machining time.

**Figure 12:**
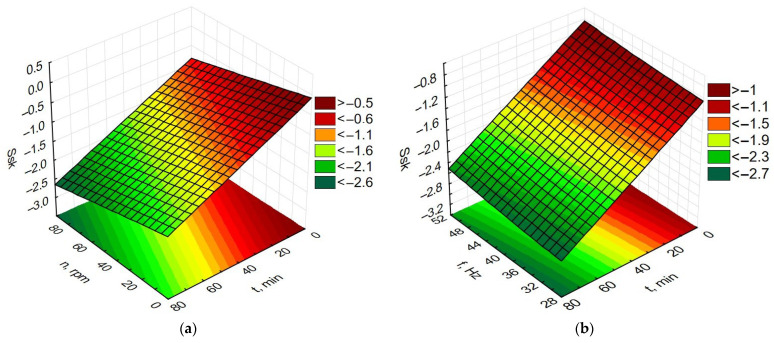
Dependence of Ssk changes (**a**) for rotational machining as a function of rotational speed and machining time; (**b**) for vibration machining as a function of frequency and machining time.

Data processing in the Statistica program allowed for the preparation of graphs of the dependence of time and rotational speed for rotary machining and the dependence of the influence of machining time and vibration frequency for vibratory machining on changes in MRR, Ra, Rp, and Ssk, as presented in Figure 9, Figure 10, Figure 11 and Figure 12.

Based on the graphs in Figure 9, it can be concluded that the greatest changes in the amount of material removed per unit of time, MRR, occur in the first minutes of both rotary and vibration machining. Extending the processing time causes a reduction in the MRR change value, achieving a minimum efficiency of approximately 60 min of processing. Extending machining times beyond 60 min increases the MRR changes. Comparing Figure 9a,b shows that larger MRR changes are observed for rotary machining. Therefore, for finishing processes that are limited by machining time, rotary machining can be used at high drum speeds as well as vibration machining.

Based on Figure 6 and the graphs in Figure 11 showing changes in the arithmetic mean deviation of the roughness profile from the mean line Ra as a function of vibration duration, rotational speed, and frequency, it can be concluded that the surface without the treatment is characterized by a value of the parameter Ra = 8.5 µm. The shape of the graphs for both treatments is slightly different. Similar to both rotary and vibratory machining, the Ra parameter decreases with the duration of the container machining process. The key difference, however, is that for rotary machining, the Ra parameter changes are influenced by rotational speed, while for vibratory machining, the vibration frequency has no effect on the Ra parameter changes. Analyzing Figure 11a, it can be concluded that the greatest changes in Ra can be obtained for the tank rotational speed of about 50 rpm. It should be emphasized that rotational speed depends on process factors such as the size and mass of the workpieces, the size and type of the shapes, the amount of auxiliary fluids, the filling level of the machine’s working tank, and, above all, the consistency of the feedstock. Both vibration and rotational machining allow for a more than two-fold reduction in the Ra SGP parameter value, from 8.5 µm to below 3 µm after 75 min of machining.

The measurements of the geometric structure of the sample surface before the container treatment showed that the surface was characterized by a surface skewness parameter value of Ssk of about 5. Low values of the Ssk parameter indicate surfaces with numerous sharp peaks. Figure 12a,b are very similar, showing that increasing the rotational and vibration machining time increases the surface skewness parameter Ssk, reaching -2 around 60 min of machining time. This indicates the beneficial use of rotational and vibration machining to smooth the surface of protruding peaks.

The high efficiency of the rotational machining process results from the high rotational speeds used for the tests. Literature data [31] suggest using rotational speeds below 23.2 rpm for a tank with a diameter as in the test case, D = 0.32 m. Such high rotational speeds cause intense interactions between abrasive media and the workpieces being tested. It is also crucial to consider the consistency of the working charge [32], which depends on the amount of supporting fluids. A possible perspective for further research could be to assess the impact of the amount of media, the filling level of the working container, and the consistency of the charge.

**Figure 13:**
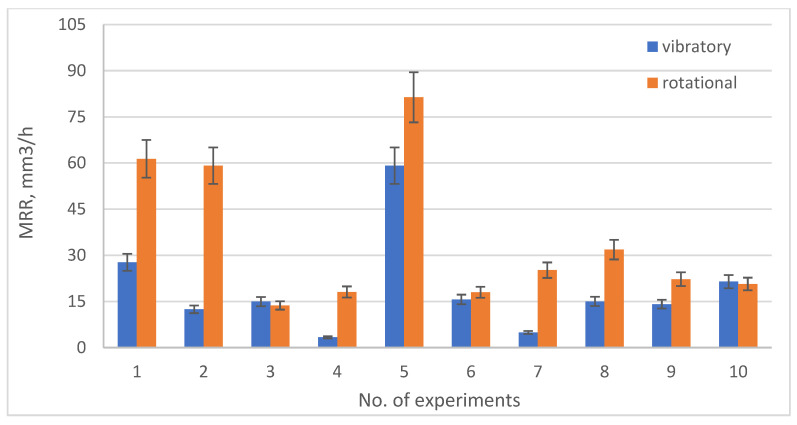
Graph of MRR losses depending on the type of treatment and experiment no.

**Figure 14:**
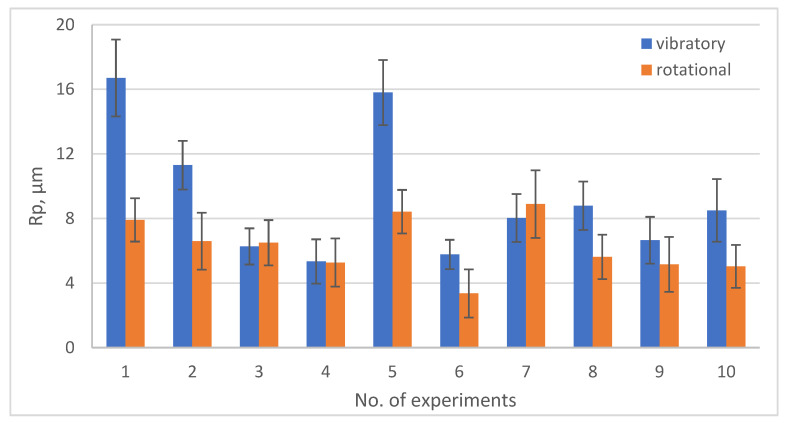
Graph of Rp changes depending on the type of treatment and experiment no.

**Figure 15:**
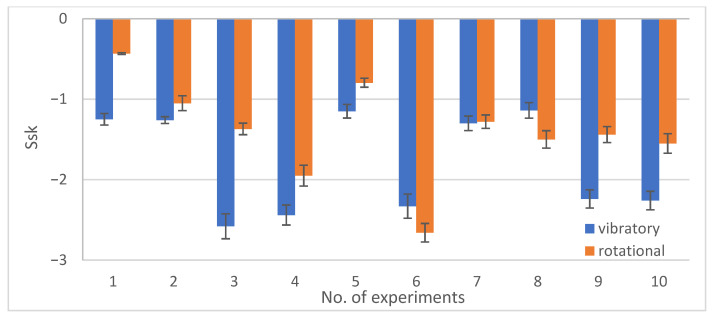
Graph of Ssk changes depending on the type of treatment and experiment no.

**Figure 16:**
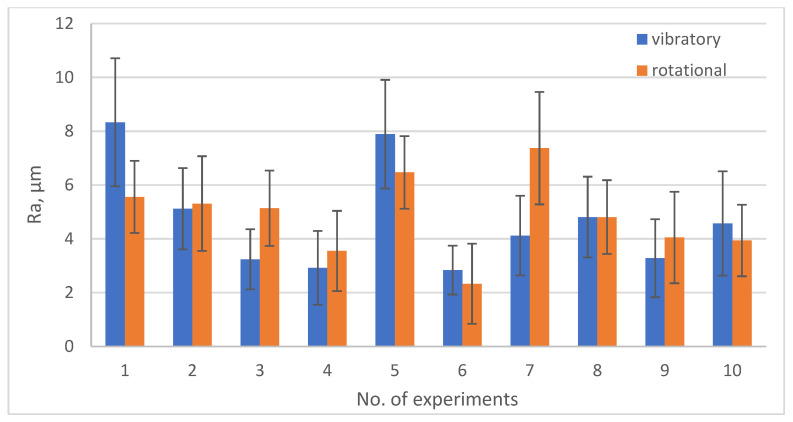
Graph of Ra changes depending on the type of treatment and experiment no.

Figure 13, Figure 14, Figure 15 and Figure 16 show the changes in MRR, Rp, Ssk, Ra depending on the type of machining (rotary and vibratory) and the experiment number.

Analyzing Figure 13, it can be observed that only in experiments 3 and 10, vibratory machining is characterized by a slightly larger decrease in MRR compared to rotational machining. This may be due to the fact that the use of low rotational speeds (below *n* critical) in rotational machining results in identical MRR decreases as for vibration machining and low vibration frequencies.

Looking at the Rp values for individual experiment numbers, it can be seen that only in experiments 3 and 7 were lower Rp values obtained for vibratory machining. In the remaining cases, significantly better results, with lower values of the maximum height of the highest elevation (peak) of the roughness profile, measured from the mean Rp line, were obtained for rotary machining. Experiments 3 and 7 were conducted at low drum rotational speeds during rotary machining, 20.8 and 11.2 rpm, respectively. These values did not exceed the critical speed of approximately 23.2 rpm recommended in the literature [31], hence the low efficiency of rotary machining.

Analyzing Figure 15, the surface skewness Ssk plot, depending on the machining type and experiment number, it can be concluded that surface skewness values closer to zero were obtained in most cases for rotational machining. Values close to zero indicate that the height distribution is symmetrical about the mean plane. The lower the values, the more valleys than peaks the surface contains. Only in experiments 6 and 8 were values closer to zero obtained for vibratory machining. This situation may result from the fact that excessively high rotational speeds, such as experiment 8–81.6 rpm, cause intense interactions and strong media impact on the workpieces, which translates into severe surface scratching. A similar situation may occur in the case of excessively long machining time, such as experiment no. 6, which lasted 75 min. Vibratory machining is performed at the maximum vibration frequency or for the longest machining time allowed to obtain a surface with less surface skewness than rotary machining.

Looking at the Ra values for individual experiment numbers, it can be seen that in the case of experiments no. 2, 3, 4, 7, 8, and 9, lower Ra values were obtained for vibratory machining. In the remaining cases, significantly better results, with lower values of the arithmetic mean deviation of the roughness profile from the mean Ra line, were obtained for rotary machining. This situation is determined by the fact that high rotational speeds in the case of rotational machining, as already mentioned, cause intense interactions and strong media impacts on the workpieces, which translates into severe surface scratching. Also, very low rotational speeds of 11.2 rpm, as in experiment no. 7, do not allow for achieving the effects of reducing the Ra parameter, as in the case of vibratory machining. This situation is determined by the fact that high rotational speeds in the case of rotational machining, as already mentioned, cause intense interactions and strong media impacts on the workpieces, which translates into severe surface scratching. Also, very low rotational speeds of 11.2 rpm, as in experiment no. 7, do not allow for achieving the effects of reducing the Ra parameter, as in the case of vibratory machining.

Experiments carried out at standard recommended rotational speed settings during rotational machining translate into higher amplitude parameters of surface roughness, such as Ra, Rp, etc. The novelty of this work is the use of significantly higher rotational speeds for rotational machining than previously recommended. The results clearly confirm that high-speed rotational machining enables machining results comparable to, or even significantly better than, vibratory machining. This new approach to high-speed rotational machining allows potential users to achieve the desired surface geometric structure parameters and mass losses in a significantly shorter time. This time reduction will translate into increased efficiency of finishing operations, thus reducing the machining costs per part. However, caution should be exercised when using increased rotational speeds for two reasons. Adding fluids to aid processing can cause geometric deformation of delicate workpieces made of soft materials or with thin walls. Processing without fluids or lubricants and using excessively high rotational speeds prevents the media from flowing due to centrifugal force and causes it to adhere to the inner walls of the processing container. When using high-speed rotational machining, the machining time and rotational speeds should be individually selected each time, taking into account the size of the container used, the type of media (size, shape, type), the amount of cutting fluids and, above all, the amount of the material being processed, its type, size and the material from which they are made.

## 4. Conclusions

The conducted research allows for a comparison of the effective amount of material removed per unit of time (MRR) and changes in surface geometric structure parameters (Ra, Rp, and Ssk) for rotary and vibration machining. Based on the experimental studies, mathematical models were developed as a function of machining time, rotational speed, and vibration frequency. For the assumed significance level of α = 0.05, the developed mathematical models are characterized by high and very high values of the correlation coefficient R.

The use of high rotational speeds in rotational machining allows for results comparable to, or even better than, vibratory machining.

The mathematical models and graphs obtained demonstrate that in the vast majority of cases considered, machining time has the greatest impact on the results of container machining.

The largest changes in the amount of material removed per unit of MRR time occur in the initial stages of both rotary and vibration machining. Increasing machining time reduces the value of the MRR change.

Comparing the changes in MMR for rotary and vibratory machining, it can be concluded that greater changes in MRR are observed for rotary machining.

Analyzing the changes in the arithmetic mean deviation of the surface unevenness height from the reference line Ra as a function of the duration, rotational speed, and frequency of vibrations, it can be stated that for vibratory machining, it depends only on the machining time, while for rotational machining, it depends on the time and frequency of the container vibrations.

Both vibration and rotational machining allow the Ra parameter to be reduced by more than half, from 8.5 µm to below 3 µm after 75 min of loose abrasive machining. However, it should be emphasized that the best Ra reduction was achieved with 54 rpm rotary machining for 75 min, which amounted to 2.33 µm.

Isomer views and results obtained from surface geometric structure measurements confirm that both rotational and vibrational machining are excellent for smoothing the surface of sharp surface irregularities. After 75 min of rotational and vibrational machining, the surface kurtosis parameter Ssk was nearly halved.

## Figures and Tables

**Table 1:** Parameters and results of vibratory and rotational machining during the experiment.

	No.	t, min	f, Hz	MRR, mm^3^/h	Ra, µm	Rp, µm	Ssk
Vibratory machining	1	15	33	27.7	8.33	16.7	−1.25
	2	15	47	12.4	5.12	11.3	−1.26
	3	65	33	14.9	3.24	6.27	−2.58
	4	65	47	3.4	2.92	5.34	−2.44
	5	5	40	59.1	7.89	15.8	−1.15
	6	75	40	15.6	2.84	5.77	−2.33
	7	40	30	4.9	4.12	8.03	−1.3
	8	40	50	15.0	4.81	8.79	−1.14
	9	40	40	14.1	3.28	6.65	−2.24
	10	40	40	21.4	4.57	8.5	−2.26
	No.	t, min	f, Hz	MRR, mm^3^/h	Ra, µm	Rp, µm	Ssk
Rotational machining	1	15	20.8	61.3	5.56	13	−0.434
	2	15	72	59.1	5.31	10.6	−1.05
	3	65	20.8	13.7	5.14	10.8	−1.37
	4	65	72	18.1	3.55	6.74	−1.95
	5	5	54.4	81.3	6.47	13	−0.796
	6	75	54.4	18.0	2.33	4.96	−2.66
	7	40	11.2	25.2	7.37	14.4	−1.28
	8	40	81.6	31.8	4.81	9.29	−1.5
	9	40	54.4	22.2	4.05	8.52	−1.44
	10	40	54.4	20.7	3.94	7.11	−1.55

**Table 2:** Results of ANOVA variance analysis for MRR of the sample after rotational machining.

	Sum of Squares (SS)	Number of Degrees of Freedom	Mean Square	F	*p*	Influence %
Model	35,676.36	3	35,676.36	745.4	0.05	
*t*	28,946.83	1	28,946.83	604.80	0.000	81.13
*t* ^2^	6388.08	1	6388.08	133.47	0.001	17.91
*n* ^2^	341.45	1	341.45	7.13	0.037	0.96
Error	287.17	6	47.86			
Total SS	35,908.29	9	R^2^ = 0.99			R^2^-Adj = 0.99

**Table 3:** Results of ANOVA variance analysis for MRR of the sample after vibratory machining.

	Sum of Squares (SS)	Number of Degrees of Freedom	Mean Square	F	*p*	Influence %
Model	10,915.22	2	10,915.22	46.48	0.008	
*t*	7494.21	1	7494.21	29.85	0.001	68.66
*t* ^2^	3421.01	1	3421.01	16.63	0.007	31.34
Error	1754.19	7	251.03			
Total SS	12,672.41	9	R^2^ = 0.86			R^2^-Adj = 0.82

**Table 4:** Results of ANOVA variance analysis for Ra of the sample after rotational machining.

	Sum of Squares (SS)	Number of Degrees of Freedom	Mean Square	F	*p*	Influence %
Model	14.981	3	14.981	29.19	0.088	
*t*	8.032	1	8.032	15.65	0.007	53.61
*n*	3.541	1	3.541	6.90	0.039	23.64
*n* ^2^	3.408	1	3.408	6.64	0.042	22.75
Error	3.080	6	0.513			
Total SS	19.285	9	R^2^ = 0.84			R^2^-Adj = 0.76

**Table 5:** Results of ANOVA variance analysis for Ra of the sample after vibratory machining.

	Sum of Squares (SS)	Number of Degrees of Freedom	Mean Square	F	*p*	Influence %
Model	26.037	1	26.037	24.13	0.001	
*t*	26.037	1	26.037	24.13	0.001	100.00
Error	8.633	8	1.079			
Total SS	34.669	9	R^2^ = 0.75			R^2^-Adj = 0.72

**Table 6:** Results of ANOVA variance analysis for Rp of the sample after rotational machining.

	Sum of Squares (SS)	Number of Degrees of Freedom	Mean Square	F	*p*	Influence %
Model	72.58	3	72.58	63.78	0.025	
*t*	37.859	1	37.859	33.27	0.001	52.16
*n*	23.396	1	23.396	20.56	0.004	32.23
*n* ^2^	11.325	1	11.325	9.95	0.020	15.60
Error	6.829	6	1.138			
Total SS	85.186	9	R^2^ = 0.92			R^2^-Adj = 0.88

**Table 7:** Results of ANOVA variance analysis for Rp of the sample after vibratory machining.

	Sum of Squares (SS)	Number of Degrees of Freedom	Mean Square	F	*p*	Influence %
Model	116.933	1	116.933	30.24	0.001	
*t*	116.933	1	116.933	30.24	0.001	100.00
Error	30.934	8	3.867			
Total SS	147.867	9	R^2^ = 0.79			R^2^-Adj = 0.76

**Table 8:** Results of ANOVA variance analysis for Ssk of the sample after rotational machining.

	Sum of Squares (SS)	Number of Degrees of Freedom	Mean Square	F	*p*	Influence %
Model	2.881	2	2.881	42.16	0.05	
*t*	2.495	1	2.495	36.51	0.001	86.60
*n*	0.386	1	0.386	5.65	0.049	13.40
Error	0.478	7	0.068			
Total SS	3.360	9	R^2^ = 0.86			R^2^-Adj = 0.82

**Table 9:** Results of ANOVA variance analysis for Ssk of the sample after vibratory machining.

	Sum of Squares (SS)	Number of Degrees of Freedom	Mean Square	F	*p*	Influence %
Model	2.875	2	2.875	37.88	0.021	
*t*	2.187	1	2.187	28.82	0.001	76.07
*f* ^2^	0.688	1	0.688	9.06	0.020	23.93
Error	0.531	7	0.076			
Total SS	3.406	9	R^2^ = 0.84			R^2^-Adj = 0.80

**Table 10:** Regression equations of rotational and vibratory machining.

The Regression Equation		R	R^2^	R^2^-Adj
Rotational	MRR = 247.18 – 6.83*t* + 0.055*t*^2^ + 0.002*n*^2^	0.99	0.99	0.99
	Ra = 9.94 – 0.04*t* – 0.15*n* + 0.001*n*^2^	0.92	0.84	0.76
	Rp = 20.64 – 0.09*t*−0.29*n* + 0.02*n*^2^	0.96	0.92	0.88
	Ssk = 0.08 – 0.02*t* – 0.009*n*	0.93	0.86	0.82
Vibratory	MRR = 155.93 – 4.45*t* + 0.04*t*^2^	0.93	0.86	0.82
	Ra = 7.61 – 0.07*t*	0.87	0.75	0.72
	Rp = 15.46 – 0.15*t*	0,89	0.79	0.76
	Ssk = 1.15 – 0.02*t* + 0.0001*f*^2^	0.92	0.84	0.80

## Data Availability

The original contributions presented in this study are included in the article/Supplementary Materials. Further inquiries can be directed to the corresponding author.

## Supplementary Materials

The following supporting information can be downloaded at: https://www.mdpi.com/article/10.3390/ma19081554/s1, Figure S1. Residual analysis for the MRR model of rotational machining (a) normal plot of residuals; (b) residuals relative to predicted values; (c) dependence of the approximated value on the value from experiment.; Figure S2. Residual analysis for the MRR model of vibratory machining (a) normal plot of residuals; (b) residuals relative to predicted values; (c) dependence of the approximated value on the value from experiment; Figure S3. Residual analysis for the Ra model of rotational machining (a) normal plot of residuals; (b) residuals relative to predicted values; (c) dependence of the approximated value on the value from experiment; Figure S4. Residual analysis for the Ra model of vibratory machining (a) normal plot of residuals; (b) residuals relative to predicted values; (c) dependence of the approximated value on the value from experiment; Figure S5. Residual analysis for the Rp model of rotational machining (a) normal plot of residuals; (b) residuals relative to predicted values; (c) dependence of the approximated value on the value from experiment; Figure S6. Residual analysis for the Rp model of vibratory machining (a) normal plot of residuals; (b) residuals relative to predicted values; (c) dependence of the approximated value on the value from experiment; Figure S7. Residual analysis for the Ssk model of rotational machining (a) normal plot of residuals; (b) residuals relative to predicted values; (c) dependence of the approximated value on the value from experiment; Figure S8. Residual analysis for the Ssk model of vibratory machining (a) normal plot of residuals; (b) residuals relative to predicted values; (c) dependence of the approximated value on the value from experiment.
