# Supplementary material for: Comparative Studies of the Effectiveness of Rotational and Vibratory Machining
Source: Materials (Basel). 2026 Apr 13;19(8):1554. doi: 10.3390/ma19081554 (PMC13118132; doi:10.3390/ma19081554)
Supplement: Supplementary file 1 [file materials-19-01554-s001.zip › materials-4213172-supplementary.pdf]

materials

Article

Comparative Studies of the Effectiveness of Rotational and Vibratory Machining

Damian Bańkowski *, Piotr Młynarczyk and Wojciech Depczyński

Department of Materials Science and Materials Technology, Faculty of Mechatronics and Mechanical Engineering, Kielce University of Technology, al. 1000-lecia P.P. 7, 25-314 Kielce, Poland; piotrm@tu.kielce.pl (P.M.); wdep@tu.kielce.pl (W.D.)
* Correspondence: dbankowski@tu.kielce.pl

Additionally, the correctness of the developed models was verified by residual analysis. The residual is defined as the difference between the experimental value and the RSM model value [43]. Residual values equal to or close to zero indicate a correctly developed model [44, 45]. Residual values reaching values of several dozen percent of the experimentally obtained values indicate an incorrectly developed model.

Analyzing the graphs of the expected value versus residuals – Figures S1a-S8a – reveals a linear relationship. This linear relationship indicates that the developed mathematical models were correctly developed as a function of time, frequency, and rotational speed of container processing.

The dependence of residuals as a function of predicted values (from the model developed using the RMS method) presented in Figures S1b-S8b indicates the random nature of the residuals. To graphically confirm the correctly developed mathematical models of changes in MRR, Ra, Rp, and Ssk, graphs S1c)-S8c were developed showing the dependence of the approximated value on the value obtained in actual studies. The developed graphs S1c)-S8c of residuals against the approximated value on the value obtained in actual studies showed linear dependencies with a small scatter of residual values, which confirms the correctness / high degree of fit of the developed models / confirming the adequacy of the developed regression models.

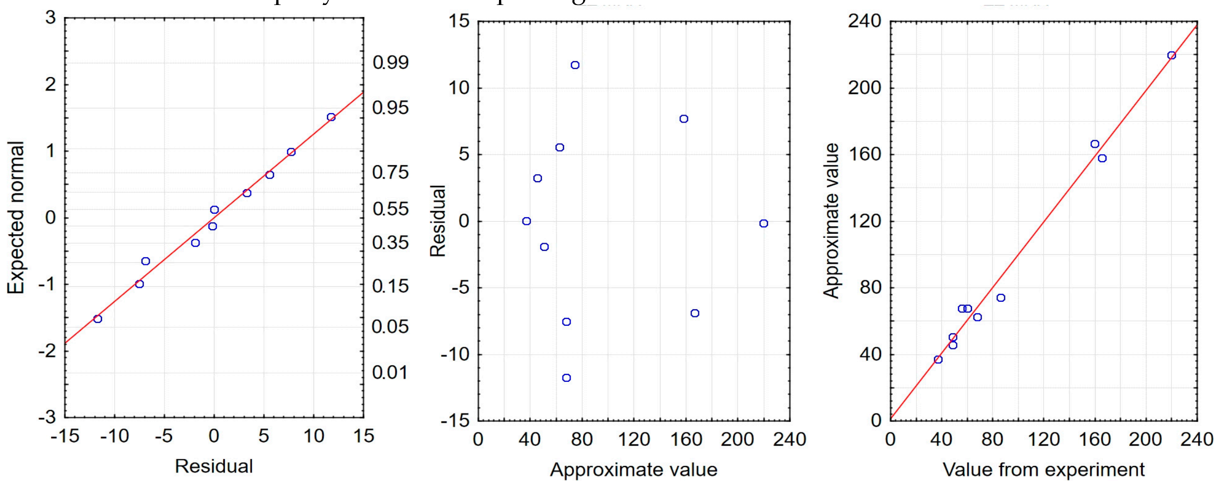

(**a**) (**b**) (c)

Figure S1. Residual analysis for the MRR model of rotational machining (a) normal plot of residuals; (**b**) residuals relative to predicted values; (**c**) dependence of the approximated value on the value from experiment.

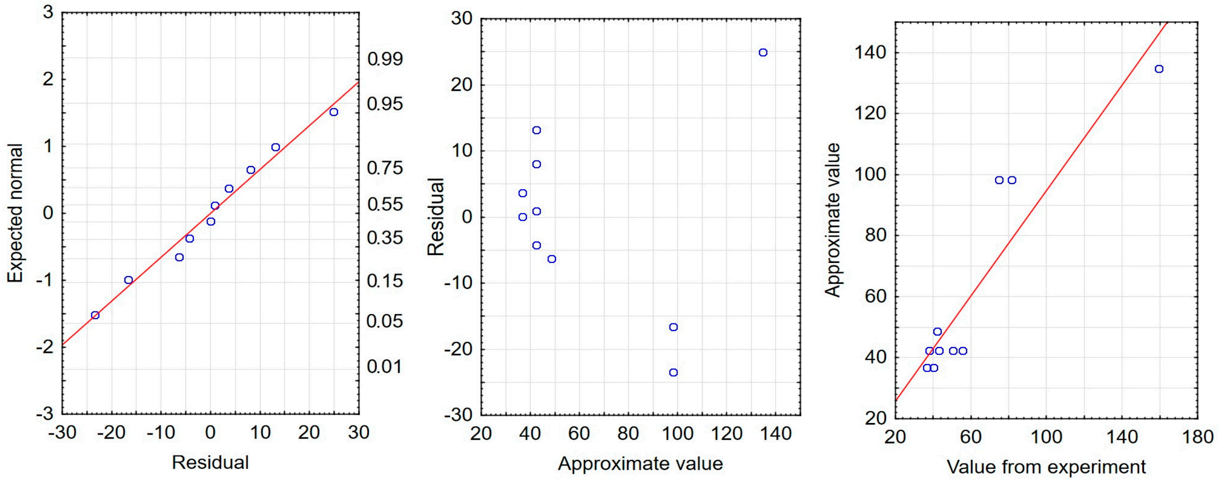

(**a**) (**b**) (c)

Figure S2. Residual analysis for the MRR model of vibratory machining (a) normal plot of residuals; (**b**) residuals relative to predicted values; (**c**) dependence of the approximated value on the value from experiment.

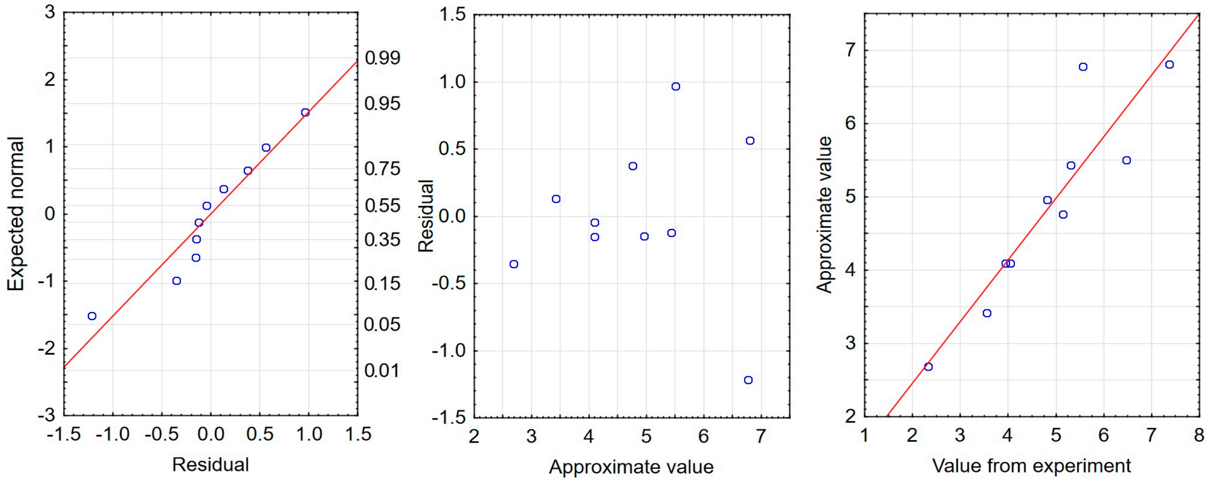

(**a**) (**b**) (c)

Figure S3. Residual analysis for the Ra model of rotational machining (a) normal plot of residuals; (**b**) residuals relative to predicted values; (**c**) dependence of the approximated value on the value from experiment.

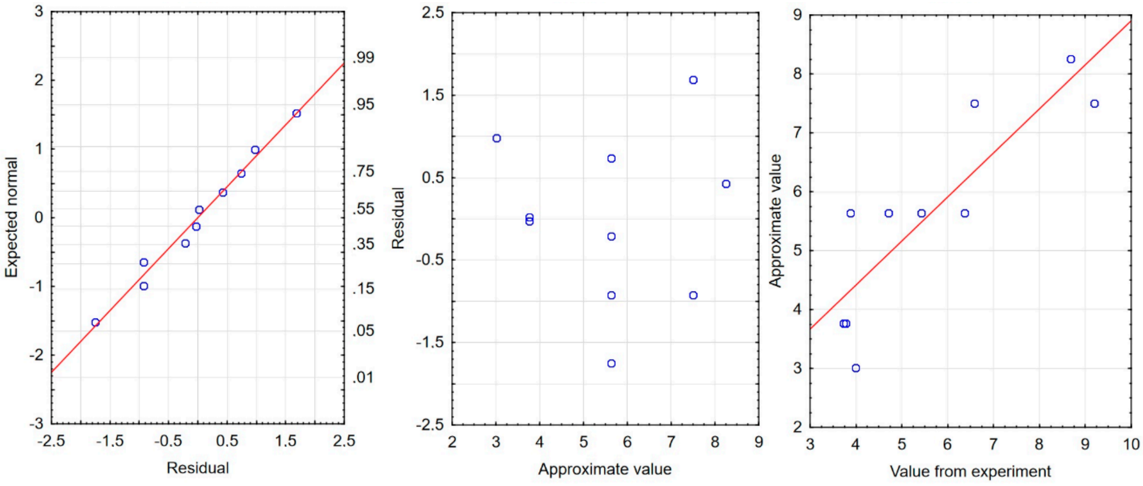

(**a**) (**b**) (c)

Figure S4. Residual analysis for the Ra model of vibratory machining (a) normal plot of residuals; (**b**) residuals relative to predicted values; (**c**) dependence of the approximated value on the value from experiment.

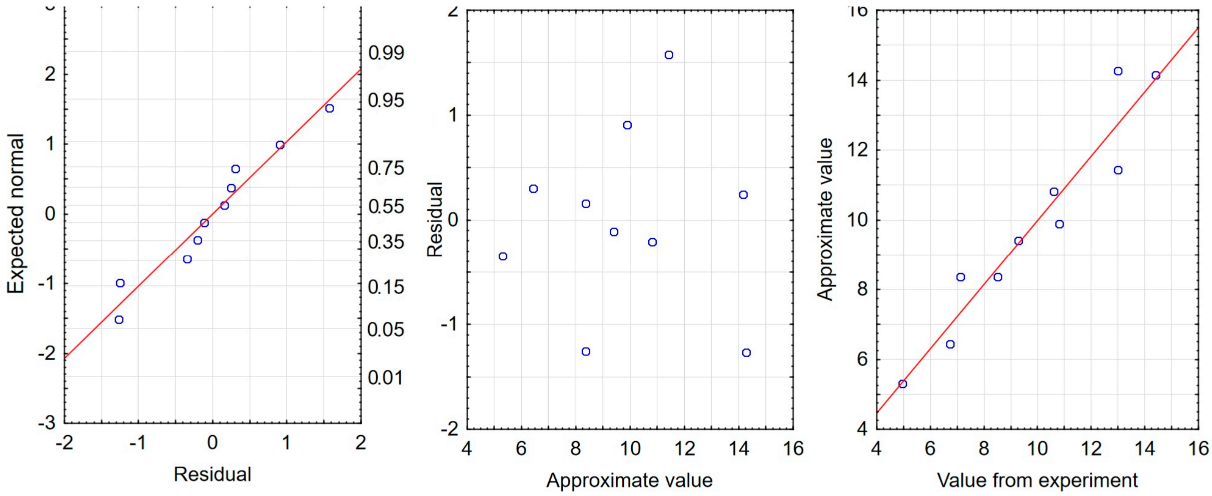

(**a**) (**b**) (**c**)

Figure S5. Residual analysis for the Rp model of rotational machining (a) normal plot of residuals; (**b**) residuals relative to predicted values; (**c**) dependence of the approximated value on the value from experiment.

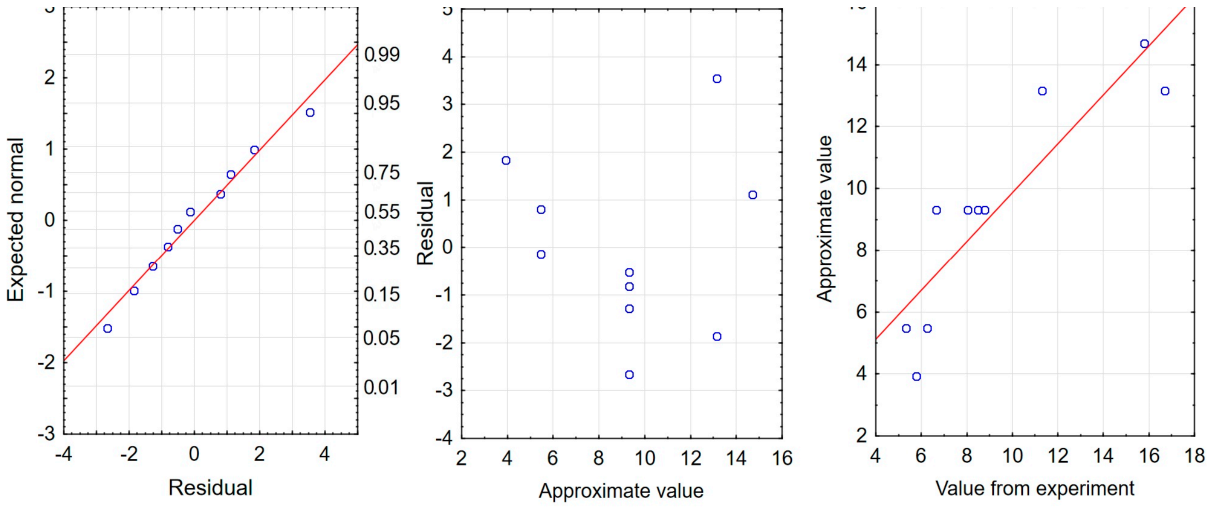

(a) (**b**) (c)

Figure S6. Residual analysis for the Rp model of vibratory machining (a) normal plot of residuals; (**b**) residuals relative to predicted values; (**c**) dependence of the approximated value on the value from experiment.

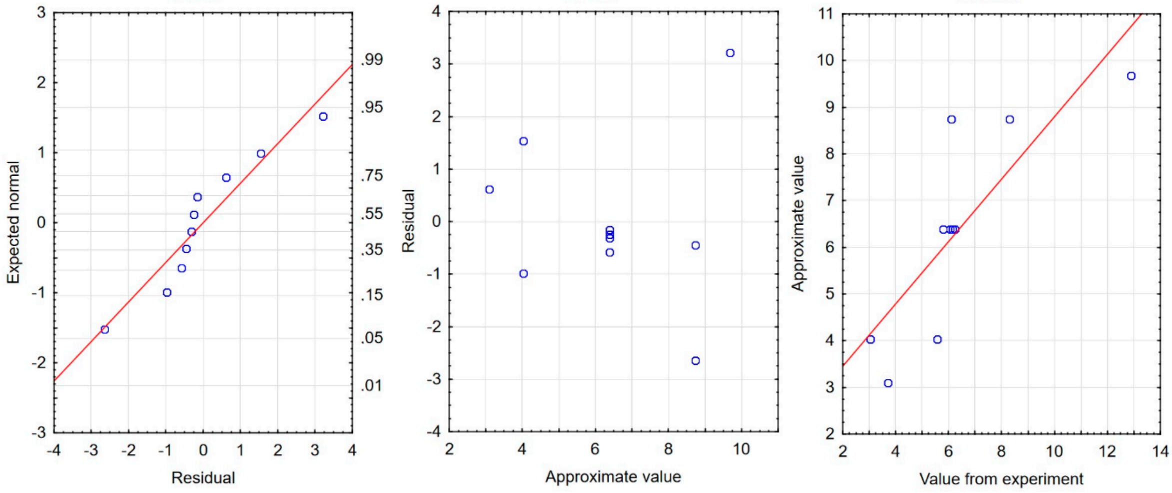

(a) (b) (c)

Figure S7. Residual analysis for the Ssk model of rotational machining (a) normal plot of residuals; (**b**) residuals relative to predicted values; (**c**) dependence of the approximated value on the value from experiment.

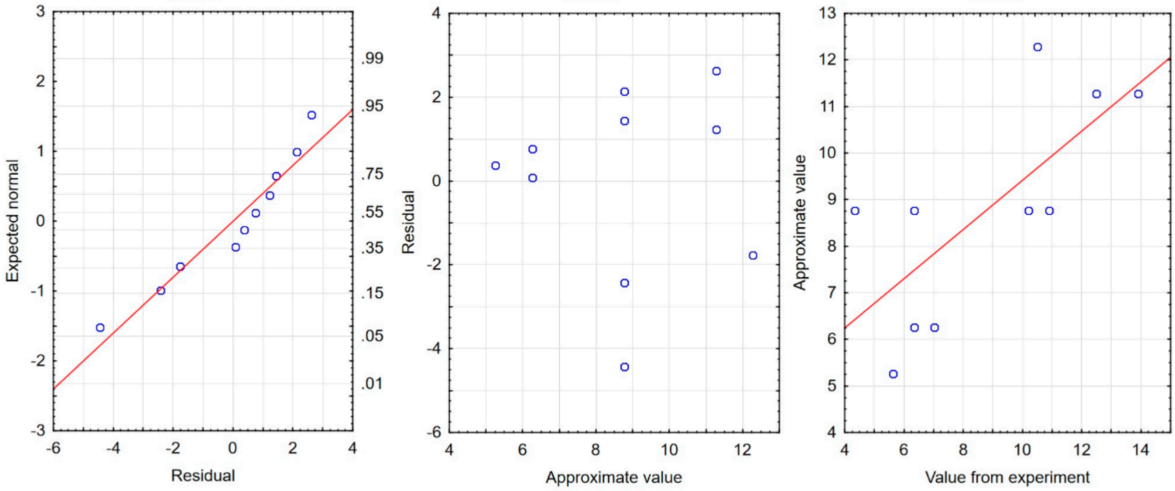

(**a**) (**b**) (c)

Figure S8. Residual analysis for the Ssk model of vibratory machining (a) normal plot of residuals; (**b**) residuals relative to predicted values; (**c**) dependence of the approximated value on the value from experiment.
